# Retraction: Histone methyltransferase SETD1A induces epithelial-mesenchymal transition to promote invasion and metastasis through epigenetic reprogramming of snail in gastric cancer

**DOI:** 10.3389/fcell.2025.1639674

**Published:** 2025-06-13

**Authors:** Frontiers Editorial Office

The journal retracts the 07 June 2021 article cited above.

Following publication, concerns were raised regarding the integrity of the images in the published figures. The authors failed to provide a satisfactory explanation during the investigation, which was conducted in accordance with Frontiers’ policies.

This retraction was approved by the Chief Executive Editor of Frontiers. The authors received a communication regarding the retraction and had a chance to respond. This communication has been recorded by the publisher.

Frontiers would like to thank the users on PubPeer for bringing the published article to our attention.

